# *CleAir* Monitoring System for Particulate Matter: A Case in the Napoleonic Museum in Rome

**DOI:** 10.3390/s17092076

**Published:** 2017-09-11

**Authors:** Eugenio Fazio, Valerio Bonacquisti, Marta Di Michele, Francesca Frasca, Angelo Chianese, Anna Maria Siani

**Affiliations:** 1 Department of Fundamental and Applied Science for Engineering, Sapienza Università di Roma, 00185 Rome, Italy; valerio.bonacquisti@uniroma1.it; 2 Department of Physics, Sapienza Università di Roma, 00185 Rome, Italy; dimichele.1200132@studenti.uniroma1.it (M.D.M.); annamaria.siani@uniroma1.it (A.M.S.); 3 Department of Earth Sciences, Sapienza Università di Roma, 00185 Rome, Italy; f.frasca@uniroma1.it; 4 Department of Chemical Engineering Materials and Environment, Sapienza Università di Roma, 00185 Rome, Italy; angelo.chianese@uniroma1.it

**Keywords:** particulate matter, PM10, spectroscopy, gravimetry, light scattering, optics

## Abstract

Monitoring the air particulate concentration both outdoors and indoors is becoming a more relevant issue in the past few decades. An innovative, fully automatic, monitoring system called *CleAir* is presented. Such a system wants to go beyond the traditional technique (gravimetric analysis), allowing for a double monitoring approach: the traditional gravimetric analysis as well as the optical spectroscopic analysis of the scattering on the same filters in steady-state conditions. The experimental data are interpreted in terms of light percolation through highly scattering matter by means of the stretched exponential evolution. *CleAir* has been applied to investigate the daily distribution of particulate matter within the Napoleonic Museum in Rome as a test case.

## 1. Introduction

The Paris Climate Agreement of 3 September 2016 contains four major commitments for the signing states, one of them being to stop increasing the greenhouse gas emissions as soon as possible. The term “greenhouse gas” refers to carbon dioxide, methane, particulate matter, nitrous oxide and ozone. Their control could slow global warming while at the same time improving public health and agricultural yield [1]. Therefore, monitoring the inhalable fine particulate in the air is a topical issue and requires a more accurate and versatile equipment in accordance with current legislation [2,3,4,5,6,7], which provides only gravimetric measurements. This technique is subject to rigorous standards to limit errors and the influence on the weight of aerosols, made up of noncombustible water or hydrocarbons. It has numerous disadvantages—first of all the time lag between sampling on filters and the determination of deposited particulate matter, which can also be very relevant at the daily time scale. On the other hand, it has the capability of sampling air on physical media that can be stored, promoting the creation of air databases for postprocessing. Other techniques have been developed parallel to the gravimetric one to speed up the measurement: the nephelometric of diffused light at fixed angles, usually at 90° from gaseous samples containing particulate matter; scattering spectroscopy; beta beam attenuation; optical absorption or aethalometry. Scatter-based techniques are usually very fast and allow to monitor particulate matter in real time; on the contrary, they are usually unclear and incur serious measurement errors [8,9,10,11]. The beta radiation attenuation is a well-established technique with many commercial devices available, but it has never been officially adopted as a monitoring technique for the use of ionizing radiation.

The measurement of optical attenuation, the aethalometer, appeared for the first time in 1984 [12], mainly to measure the fraction of black carbon present in particulate matter. Later on, aethalometry was used in a spectroscopic manner [13] in order to evaluate the optical absorbance, which was demonstrated to scale as one-half of the optical depth instead of linearly as stated by the Beer’s law. Aethalometry uses long strips of paper to collect dust, with an automatic movement of the paper when dust gives an exponential decreasing of the transmission as high as 25% of the initial value. Consequently, aethalometers always analyze transient regimes, with rapid variations due to the exponential trend.

In order to overpass such undesirable behavior, we have considered the reverse possibility to analyze filters in their stationary regime, i.e., when the amount of dust reaches almost a steady state, by means of *CleAir*. *CleAir* is a fully automatic system that samples the air on quartz fiber filters, according to EN 12341:2014 [6] and measures them in situ using a spectroscopic optical transmission technique using six visible wavelengths (455, 470, 528, 590, 617 and 625 nm). It was designed and built by the Department of Fundamental and Applied Science for Engineering in Sapienza University in Rome [14]. *CleAir* measurement is similar to the aethalometric [13] technique but it is performed in order to focus the attention on the attenuation connected with the scattering. The gravimetric and the optical techniques ensure a cross-check of the sampled filters, which could be very important in Italy or in Europe where the only legally recognized technique is the use of gravimetrics. *CleAir* both provides a fast optical analysis of the air quality and makes the 24-h filters available for gravimetric measurements according to the European standard [6]. Therefore, a calibration of the optical data performed during short temporal ranges is possible in comparison with 24-hour filters. The total flexibility of *CleAir* lets the device to carry on sampling on both long (24 h) and short (4 h) temporal ranges, in order to monitor the daily evolution of PM10, (i.e., particulate matter with aerodynamic diameters that are generally 10 μm and smaller) in terms of mass concentration and average size distribution. 

The aim of this work is to present *CleAir* and to apply it in a real environment, the Napoleonic Museum in Rome. For this purpose, we have performed both daily and weekly monitoring campaigns and SEM/EDS (scanning electron microscope with energy dispersive X-ray spectroscopy) analysis of the indoor PM10.

The control of environmental parameters involved in the artworks’ deterioration processes is essential to preserve artworks and collections in museums, galleries and churches. In the last decade, monitoring campaigns of the main environmental parameters have been becoming a common practice to define the historic microclimate to which vulnerable objects to temperature and humidity changes have adapted [15], because departures from historic conditions might be risky. In recent years, it has paid to be aware of the problem represented by the atmospheric particulate deposition on artifacts in museums [16,17,18,19,20,21,22].

Some investigations have shown there is a significant increase of the particles with a diameter greater than 1 μm during the museum’s opening hours [23,24,25,26]. In this work, a study of PM10 was carried out within the Napoleonic Museum in Rome. It is housed on the ground floor of Palazzo Primoli (an historic building of 16th century), in the centre of Rome, at the crossing between Lungotevere Tor di Nona and Via Giuseppe Zanardelli (Lat. 41.9° and Long. 12.5°). Such an area is just the boundary of a limited traffic zone; consequently, significant fluxes of vehicles take place right outside the opening doors of the museum throughout the day. The museum consists of 12 rooms and preserves an important collection of paintings, original manuscripts, Napoleonic relics and family mementos donated to the city of Rome by Count Giuseppe Primoli, a descendant of the Bonaparte family. The museum is mainly visited in the winter period during the morning. The number of visitors is limited, up to few hundred per hour. Some temporary exhibitions are also scheduled during the year.

The rooms are equipped with a central heating system that was added to the original building and consists of cast iron radiators which are switched on from November until April. A humidification system is always operating in many rooms, with the aim to guarantee a range of relative humidity between 40% and 60%.

## 2. Methods

### 2.1. CleAir System

**Figure 1:**
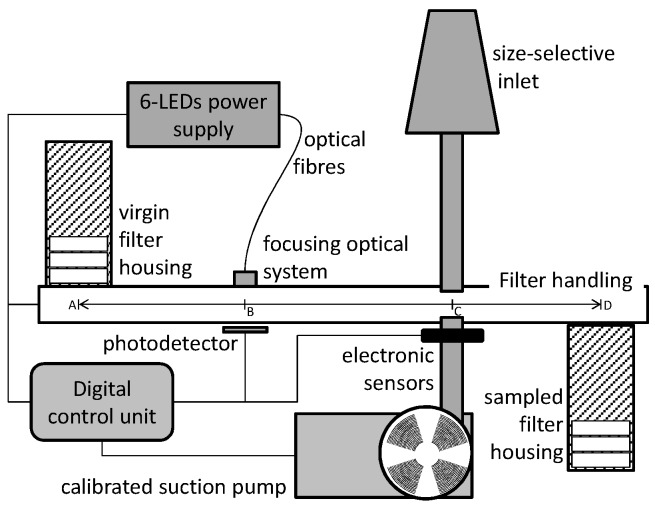
Schema of *CleAir* apparatus.

*CleAir* is a fully automatic sampling and measuring unit that collects PM10 on standard [2] circular quartz fiber filters. The *CleAir* system measures PM10 concentration and average size distribution by means of a spectroscopic light transmission analysis. Its scheme is shown in Figure 1. Each filter, after being preliminary weighted [6], is held inside a Teflon box with a metallic net in order to prevent filter deformation or breakage due to air flow [6]. An electronic control unit drives the handling system to pick up one filter box from the virgin housing and to carry it just below the optical system. Here, six optical fibers carry the light generated by six equivalent LEDs from the power-supply unit down to the filter location. The six LEDs cover the spectral bands deep blue (central wavelength 455 nm), blue (c.w. 470 nm), green (c.w. 528 nm), yellow (c.w. 590 nm), amber (c.w. 617 nm) and red (c.w. 625 nm); a reference detector monitors their light-emission stability within the power supply unit. An optical system collects the light emerging from the fibers and focuses it onto the filter. A photodetector records the light transmitted through the filter and sends such information to the general control unit. Now the filter is carried to the air sampling area, just below the inlet pipe, where a specific sealing system avoids any pressure loss from the pipe.

Ambient air passes through a size-selective inlet at a constant flow rate of 2.3 m^3^/h, using the inertial separation principle. The filter remains in such position for the sampling time and then it is transported back to the optical unit where the spectroscopic transmission is again recorded and processed by the control unit. Such procedure is repeated back and forth following the programmed sampling cycle and then, at the end, the filter is discharged in the sampled filter housing. Such sampling cycles can be fully programmed by the operator, in order to get short/long/24-h measurements upon request. *CleAir* can provide several following periods of sampling of the same filter, in order to record the daily time evolution as well as the 24-h integral according to the European regulations. *CleAir* can contain enough filters to ensure up to two weeks of measurement autonomy.

### 2.2. Stretched Exponential Analysis

The optical attenuation is mainly governed by the Mie scattering through the filter fibers. Truly, the process is governed by multiple scattering events due to the dense structure of the filters, which has been investigated by several researchers in the past. A reference work on this subject was published by Ångström in 1929 [27], where he analyzed the light transmission through the atmosphere. He described such transmission using a modified Beer’s law:

(1) $$T=e^{-ABS}=e^{-\left(\beta /\lambda^{\alpha }\right)}$$

where *ABS* is the optical absorbance, β is a coefficient that describes the optical attenuation due to the scattering, λ is the light wavelength and the α exponent is inversely proportional to the average diameter of dust particles in air. Such description is absolutely coherent with the Rayleigh scattering theory for which light diffraction on very small particles scales as λ^−4^. Such Ångström description is regularly adopted by “aerosol research community” to characterize their measurements. In 1991 Bruce et al. [28] found that the soot (black carbon, BC) absorption scales as λ^−1^. It might be the consequence of multiple scattering that enhances the absorption as well. Multiple scattering was clearly investigated by Bohren in 1987 [29] for both non-absorbing and absorbing media. Such analysis was adopted by Arnott et al. in 2005 [13] to describe the data from the Reno Aerosol Optics Experiment. Such work puts together the Ångström and Bruce models factorizing the optical absorbance as the sum of an Ångström scattering term and BC absorption. Arnott observed that the aethalometer response depends on the amount of deposit on the filter; for this reason they limited its use until the signal reached 75% of the initial transmission value. At that point the aethalometer changes position, letting the light be sampled on a clean and pristine portion of the quartz sampling tape. Such procedure monitors the initial portion of the transmission signal, where the exponential function is steeper and consequently where the largest indetermination can be accumulated.

*CleAir* follows the complementary approach to sample the air for a very long period (24 h) in order to reach an almost steady state regime for the optical transmission. On the contrary, measurements on short sampled filters can still be performed by a suitable choice of the reference “white” transmission.

The light transmission through a highly dense scattering medium can be considered as a percolation of photons through a porous medium. Percolation can be considered as a light relaxation inside a highly disordered system like a quartz filter is; thus, multiple scattering regime can be expressed in terms of a stretched exponential relationship, as a function of the wavelength λ

(2) $$T=e^{-{\left(\Lambda /\lambda \right)}^{\alpha }}$$

We call Λ the percolation characteristic wavelength; please note that its α-th power is just the β parameter in the Ångström law of Equation (1). Such further factorization of the Ångström law allows a much clearer description of the phenomenon in terms of light percolation, whose description is usually performed in terms of the stretched exponential law [30]. It has to bear in mind that, contrarily to the usual negative exponential trends where the slope is in the denominator of the exponent fraction, in such a case the percolation characteristic wavelength is at the numerator of the fraction. This means that it is proportional to the amount of deposited particulate. Unlike the atmospheric aerosols [27], also the 𝛼 exponent is now directly proportional to the average diameter of the deposited particulate on filters, and will be here called the size parameter. The α and Λ coefficients can be determined from wavelength-dependent transmission measurements. 

### 2.3. CleAir Calibration

**Figure 2:**
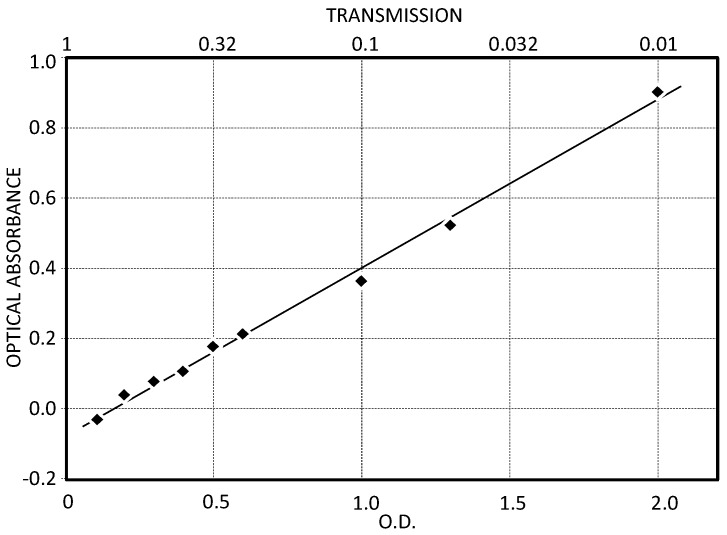
The graph shows that the optical density (O.D.) values of the calibrated neutral-density filters are linearly correlated with the measured values of the optical attenuation (absorbance) (coefficient of determination R^2^ = 0.99). Therefore, the optical head of *CleAir* maintains a good linearity down to light transmissions as low as 1% of the virgin filter ones. The negative absorbance for the lowest O.D. is a consequence of a better coupling of the input light inside each filter. The O.D., optical absorbance and transmission are dimensionless parameters.

The optical linearity of *CleAir* was analyzed using commercial neutral density filters overlapped to the quartz filters. Optical densities (O.D.) ranging between 0.1 and 2.0 were used. A good linearity for both high and low transmissions was recorded as shown in Figure 2, where the fit shows a linear regression between the measured optical absorbance and the O.D., with a coefficient of determination R^2^ equals to 0.99.

**Figure 3:**
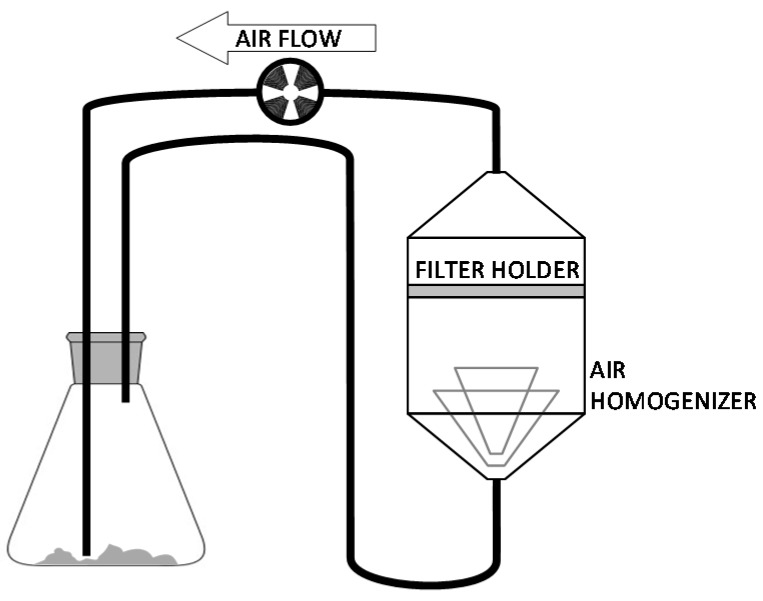
Schema of the apparatus to deposit calibrated amounts of particles on the filters.

A preliminary calibration of the optical response upon deposited particles was performed depositing defined amounts of controlled particulate on filters, using the setup shown in Figure 3. A closed circuit air-flowing system withdrew calibrated amounts of silica powder (average size 5 μm) from a flask and let them deposit on filters kept in a special holder that homogenized the air flow in order to ensure a homogeneous distribution of particles. Both flask and filters were weighted before and after the deposition process in order to determine the exact amount of deposit. The weighting procedure is described in the following paragraph.

**Figure 4:**
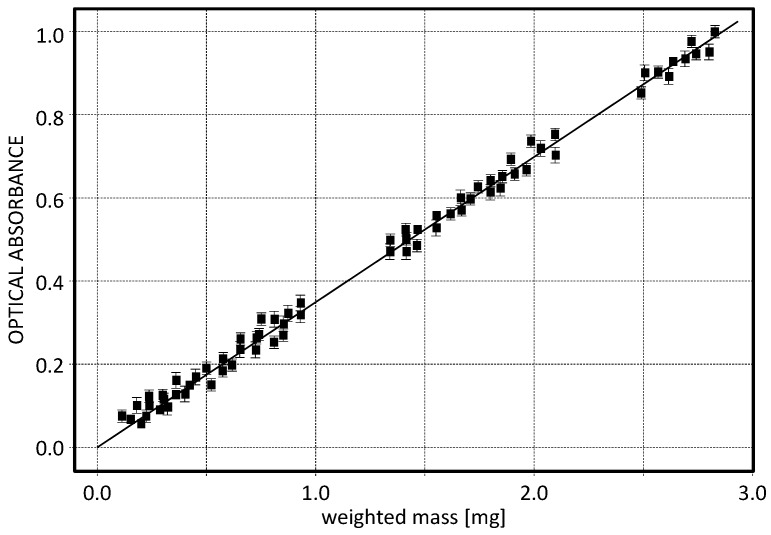
Calibration curve: optical absorbance (dimensionless parameter) vs. weighted mass. The experimental points have a coefficient of determination as high as 0.97.

Afterward, such filters were measured using the optical system of *CleAir*. In Figure 4, the optical absorbance is reported as a function of the weighted mass. From the linear fit the calibration coefficient has been derived, with a determination coefficient R^2^ of about 0.97. However it should be noted that the optical attenuation could depend on the nature of the deposited particulate which might varies from site to site. Thus, when a measuring campaign starts, a further calibration between the gravimetric and the optical masses should be performed in situ to increase the instrument sensitivity. After the second calibration the sensitivity is indeed enhanced, reaching an indetermination on the measurement of about 1–2%.

### 2.4. Standard Gravimetric Analysis

The optical analysis, performed on 24-hour filters, was compared with gravimetric analysis on the same filters, in order to have a cross-check on the amount of deposited PM10. The gravimetric measurement protocol was performed strictly according to the EN 12341:2014 standard [6], which recommends on the type of usable filters (quartz fiber filters as large as 47 mm in diameter) and on the handing procedure in order not to break them and lose mass. Filters were weighted before and after sampling to determine the mass of collected PM10, which later enabled the calculation of PM10 mass concentration. The difference between pre- and post-sampling filter weights was used to determine the ambient air mass concentration. Before weighting, each filters were conditioned at least for 48 h within a climate chamber with controlled temperature and relative humidity (i.e., 20 ± 1 °C and 50 ± 5%). The gravimetric analysis was done on a microbalance (Mettler Toledo XSD3DU with 1 μg readability) equipped with AntiStatic Kit for the neutralization of electrostatic charge. The masses of the individual weighing room blank filters were recorded at each weighing session, to check and ensure constant conditions in the weighing room, and to estimate any effect affecting the mass of the filters [8]. A final uncertainty of ±4 μg was determined for the gravimetric measurements.

### 2.5. Monitoring Campaigns within the Museum

**Figure 5:**
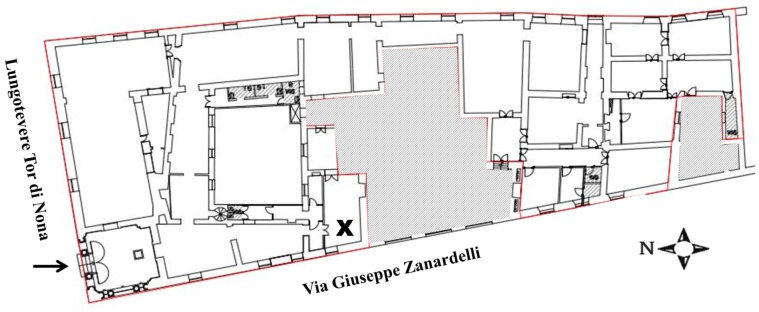
Plan of the Napoleonic Museum: the arrow refers to the entrance while the X locates the *CleAir* system. Such location was chosen to be enough internal in the museum, but relatively close to the main entrance to be affected by external conditions.

The *CleAir* system was installed in one room close to the entrance, with a window facing Via Giuseppe Zanardelli (Figure 5).

This room was chosen in an intermediate position between the inner rooms and the main entrance. The one-week measurement campaigns were carried out in the period between 15 January 2016 and 15 May 2016 (Table 1), i.e., both during winter and spring seasons. Rome has a Mediterranean climate with cool winters and warm to hot summers. Rainfall occurs mostly in winter and autumn, with a predominance of southern and western winds.

## 3. Results

### 3.1. Percolation Characteristic Wavelength

**Figure 6:**
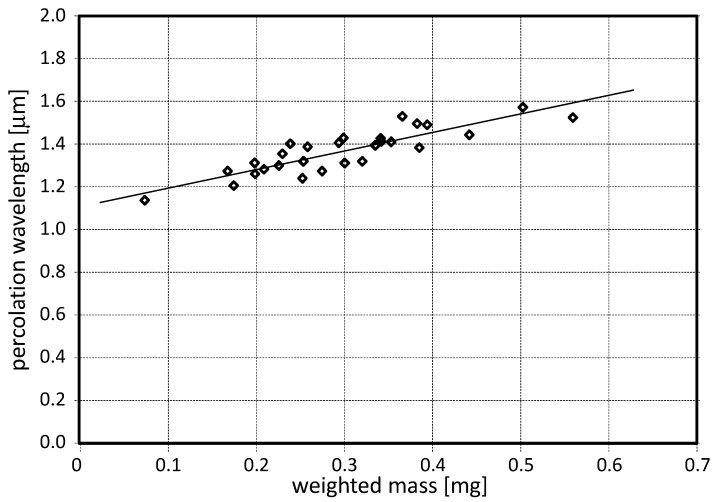
Percolation characteristic wavelength as a function of the weighted mass for the whole sampling campaign in the museum. The linear fit has a coefficient of determination R^2^ as high as 0.80. Such a plot provides a fine calibration for retrieving the mass data from the optical ones.

**Figure 7:**
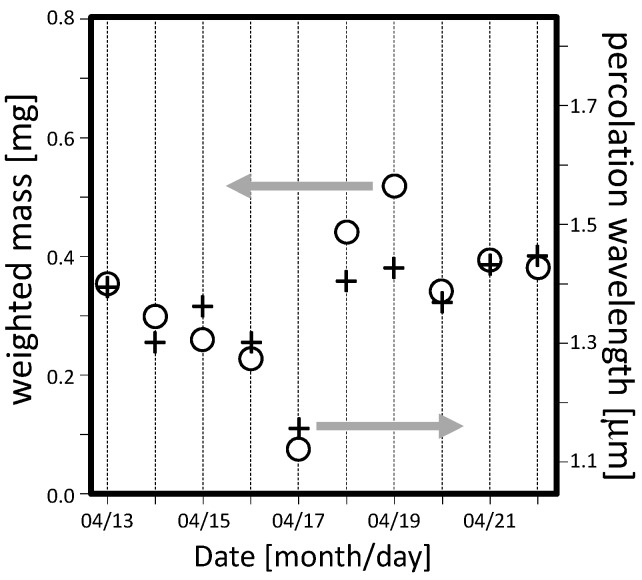
Daily PM10 concentration obtained using the optical (crosses) and the gravimetric (circles) techniques, for the campaign 13–22 April 2016. The two point sets almost overlap everywhere but on 18–19 April. From the environmental monitoring, it was pointed out that such two days had an anomalous amount of humidity that we believe affected the gravimetric measurements but not the optical ones.

The performed optical measurements show a good linearity of the percolation characteristic wavelength Λ to the amount of particulate mass, as shown in Figure 6. The linear fit has a R^2^ determination coefficient as high as 0.80. From such fit the final local calibration allows to retrieve the deposited mass from the percolation characteristic wavelength. In Figure 7, the optical (crosses) and gravimetric (circles) measurements are reported for the campaign held in the period 13–22 April 2016. For both techniques, the measurement errors are delimited within the symbols. A good agreement between optical and gravimetric measurements was found for all days but 18–19 April (Monday and Tuesday). We believe that such discrepancy is due to the gravimetric measurements which might have suffered a systematic error due to an incomplete drying process. In fact, the local revelation of humidity in the air has shown a peak for those days. Thus, we might conclude that the weighting procedure of the filters was still affected by a residual humidity that strongly increased the overall weight.

Please note that the lowest amount of dust was detected on 17 April (Figure 7), which was Sunday and the museum was closed.

### 3.2. Daily Evolution of the PM10 Concentration

**Figure 8:**
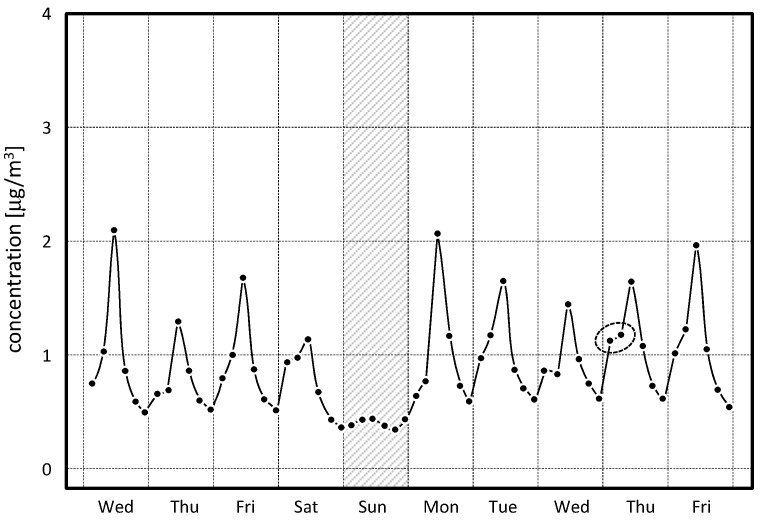
Weekly campaign on 13–22 April 2016. The daily evolution (sampled every 4 h) of the retrieved PM10 concentration points out a recurrent behaviour of the deposit, present in all the open days. Both effects describe anthropic influences on the quality of the air inside the museum. The very small fluctuation in the PM10 concentration was observed on Sunday.

*CleAir* was also set to sample air over 4 h, to optically measure the collected PM10 and then to sample again on the same filter, starting from midnight until the following midnight. At the end of the 24-h cycle, each filter was measured again and then stored to be replaced by a new one. In this way, the 24-h cycle was divided into six intermediate testing groups providing the daily evolution as well as the 24-h daily data. Typical daily trend distribution of the dust on air is reported in Figure 8 for the April monitoring campaign. Almost every day, except on Sunday, the largest deposit was recorded during the time slot between 08:00 and 12:00, and then gradually decreases starting from the following hours. This was correlated with the typical daily procedures in the museum, which report a large circulation of the internal and cleaning staff as well as the visitor flow, which is mainly concentrated during the morning, reaching several hundred units per hour. During Sunday (grey strip in Figure 8), the morning peak is absent and the particulate concentration remains practically unchanged throughout the whole day. Moreover, in the first part of the days (00:00–08:00), the amount of dust increases from the midnight values, remains almost constant during such interval (see the dotted circle in Figure 8).

This occurs because during this time interval, the internal air recirculation and heating system is turned on, increasing the convective flows and raising up again the lightest dust already deposited or in deposition by gravitational sedimentation. After 08:00, the dust in air increases, reaching its maximum values in the morning time slot (08:00–12:00). Such behavior points out an anthropic influence on the dust recirculation of the indoor air.

### 3.3. The Size Parameter α

**Figure 9:**
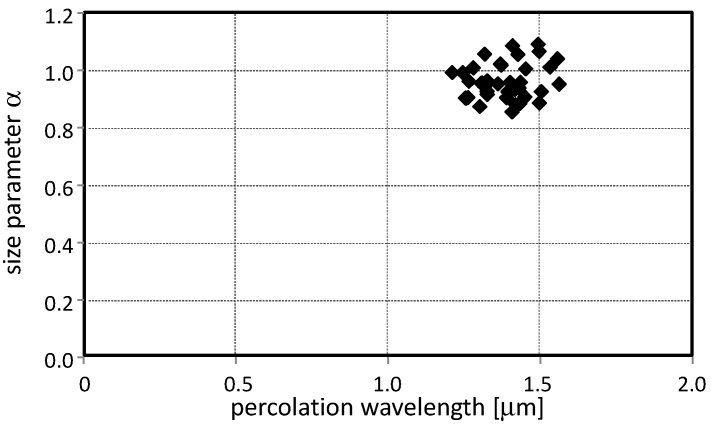
Correlation between the α size and the percolation wavelength. The small dispersion of the measures proves that the PM10 concentration in the museum varied very little with a small dispersion in size.

In all measurement campaigns, the α value varies very little, from 0.9 up to 1.1, when it is reported as a function of the percolation wavelength Λ as shown in Figure 9.

The very small dispersion of all the points proves that the PM10 in the museum air had small variations both in concentration and average size. Regarding the size, the analysis of the filters at the electron microscope allowed to characterize the real deposit of particles.

### 3.4. SEM/EDS Analysis

SEM/EDS analysis was performed using a dual-beam high-resolution field emission scanning electron microscope (model Carl Zeiss Auriga 405) [31,32] with resolution of 1 nm equipped with a Bruker QUANTAX energy dispersive X-ray spectroscopy probe. All the filter images were analyzed using particle-counting software to cross-check the visible concentration as well as the average diameter.

**Figure 10:**
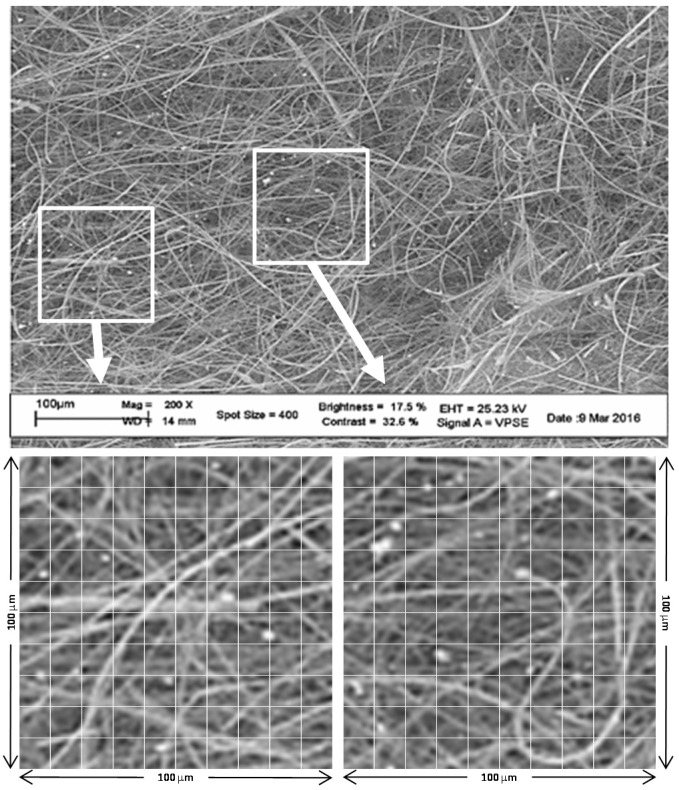
SEM image of one filter with particles. Particle size analysis from SEM images pointed out that the average particle dimension was lower than 3 μm. Large particles were rare in museum air.

From dimensional tests on SEM images (Figure 10), we have found that the average size of the collected particulate matter is ≤3 μm; particles of larger aerodynamic diameters (5 μm and 10 μm) are less abundant or even rare.

From Figure 10 it is possible to determine that the size distribution is not wide; the particles look like being almost mono-dispersed around the average size of 3 μm, as also revealed by the optical analysis in Figure 9 where the α and Λ parameters seem to be very much concentrated in a small area of the graph.

## 4. Conclusions

The *CleAir* fully automatic sampling and measuring unit was presented. *CleAir* is based on the spectroscopic analysis of the light transmission through quartz filters. Contrarily to the similar aethalometric measurements, *CleAir* works in a static condition where the filter have been stabilized in terms of particle distribution inside. This regime ensures no measurement artifacts and more precise tests. The data have been analysed in terms of light percolation through the filters by means of a stretched exponential. Such description provides a very good correlation with the gravimetric tests together with a much simpler formalism without the loss of scientific rigor. The specific tests performed within the Napoleonic Museum in Rome allowed us to point out anomalies in the daily and weekly air distribution, which is important feedback when considering management strategies. However, from the specific tests, it was found that the average concentration of PM10 in the museum remained fairly stable during all monitoring campaigns, with a mean value of 5.7 μg/m^3^ (standard deviation of 2.5 μg/m^3^)—always significantly lower than the standard threshold for outdoor PM10 (50 μg/m^3^). In particular, although it was used an aerodynamic inlet for PM10, the particulate matter detected was of a very small size, about 2.5–3.0 μm in diameter, characteristic of a PM2.5.

The *CleAir* system, thanks to the double measurement technologies, optical scattering spectroscopy and the use of gravimetrics, is very versatile and follows the required European standards for particle pollution monitoring. The comparison of the two techniques in the museum campaigns has ensured a constant measurement, with high accuracy, for both the absolute amount of particulate deposited and its granulometric composition. The data have also been confirmed by *ex-post* SEM measurements.

## Figures and Tables

**Table 1:** List of one-week measurement campaigns.

PM10 Campaigns during 2016	
15–21 January	13–22 April
6–10 February	7–15 May
14–20 February	

## Supplementary Materials

Supplementary File 1
