# Supplementary material for: CleAir Monitoring System for Particulate Matter: A Case in the Napoleonic Museum in Rome
Source: Sensors (Basel). 2017 Sep 11;17(9):2076. doi: 10.3390/s17092076 (PMC5621140; doi:10.3390/s17092076)
Supplement: Supplementary File 1 [file sensors-17-02076-s001.pdf]

DEPARTMENT OF FUNDAMENTAL AND
APPLIED SCIENCES FOR ENGINEERING

SAPIENZA
UNIVERSITÀ DI ROMA

Roma, lì September 8th, 2017

To the Editorial Office
of SENSORS

Object: submission of the revised proofs of the paper SENSORS-216518

Dear Editor

Please find enclosed the proofs of the paper SENSORS-216518 that were revised according to the comments of the editorial office. The changes are "built in track"

Here is the description of our changes: [C= your comment, A= our answer]

C1: Please carefully check the accuracy of names and affiliations. Changes will not be possible after proofreading.

A1: Names and affiliations are correct. However the email of E. Fazio has been included in the affiliation 1 .

C2: Please ensure intended meaning has been retained.

A2: OK. Brackets are included in : (i.e., particulate matter with aerodynamic diameters that are generally 10 µm and smaller)

C3: Please add the unit for the value in the figure.

A3: no units can be added because optical density (O.D.), optical absorbance and transmission are dimensionless parameters . This has been specified in the caption of the Figure

C4: Please add the unit for the value in the figure

A4: no units can be added because optical absorbance is a dimensionless parameter. This has been specified in the caption of the Figure

C5: Revised, please confirm.

A5: they are correct

C6: Please ensure intended meaning "Regarding the size"

A6: correct

C7: Incorrect ref order, 34 detected after 32. Please note that the reference should be cited in numeral order.

A7: the following reference (#33) has been cancelled "*33. Sharma, N.L.; Kuniyal, J.C.; Singh, M.; Dhyani, P.P.; Mathela, C.S.; Guleria,…………*" and the reference order has been updated

C8: Please confirm the reference type, the same as [3–8]. If it is an online source, please add the web source.
A8: The references have been updated and the order in the text has been also updated

C9: Please add publisher: city, country.
A9: done

C10: Newly added information, please confirm
A10: done

C11: Please add title and the date month year.
A11: done

Please note also that there were some typos which have been corrected as well.

With our best regards

Eugenio Fazio
